# Grading Scales of Conjunctival Inflammation

**DOI:** 10.3390/diagnostics15243200

**Published:** 2025-12-15

**Authors:** Anca-Elena Anghelache-Dobrescu, Marian-Eduard Toma, Radu-Gheorghe Bucșan, Gerhard Garhöfer, Alina Popa-Cherecheanu, Leopold Schmetterer, Alina Gabriela Gheorghe

**Affiliations:** 1 Department of Ophthalmology, ‘Carol Davila’ University of Medicine and Pharmacy, 020021 Bucharest, Romania; 2 Department of Clinical Pharmacology, Medical University of Vienna, 1090 Vienna, Austria; 3 Department of Ophthalmology, Emergency University Hospital, 050098 Bucharest, Romania; 4 Singapore Eye Research Institute, Singapore National Eye Centre, Singapore 169856, Singapore; 5 SERI-NTU Advanced Ocular Engineering (STANCE), Singapore 637553, Singapore; 6 School of Chemistry, Chemical Engineering and Biotechnology, Nanyang Technological University, Singapore 639798, Singapore; 7 Ophthalmology and Visual Sciences Academic Clinical Program, Duke-NUS Medical School, National University of Singapore, Singapore 169857, Singapore; 8 Centre for Medical Physics and Biomedical Engineering, Nanyang Technological University, Singapore 639798, Singapore; 9 Fondation Ophtalmologique Adolphe De Rothschild, 75019 Paris, France; 10 Aier Eye Hospital Group, Changsha 410005, China; 11 Clinical Institute of Ophthalmological Emergencies ‘Prof. Dr. Mircea Olteanu’, 010464 Bucharest, Romania

**Keywords:** conjunctival inflammation, hyperemia grading scales, subjective assessment, objective measurement, digital imaging, artificial intelligence, ocular surface disease, conjunctival redness

## Abstract

Conjunctival inflammation assessment is fundamental for diagnosing and monitoring various ocular surface diseases. This review summarizes grading scales available for conjunctival inflammation, discussing both subjective and objective methodologies. Widely used clinical grading systems include slit-lamp findings classification scale, Mandell scale for conjunctival injection, McMonnies and Champman-Davies scale, CCLRU (Cornea and Contact Lens Research Unit) scale, Efron scale, and VBR (validated bulbar redness) scale. They provide standardized frameworks for assessing conjunctival hyperemia and inflammation severity. However, these subjective methods are limited by inter-observer variability and lack of precision in detecting subtle changes. Recent technological advances have introduced objective digital imaging systems and automated algorithms that may offer improved reproducibility and sensitivity. Novel approaches include the integration of artificial intelligence for automated assessment. The validation of these scales across diverse patient populations has demonstrated varying degrees of reliability and clinical utility. Current evidence suggests that while traditional subjective scales remain clinically relevant, objective measurement systems provide superior repeatability and may better serve research applications requiring precise quantification of inflammatory changes. This review summarizes current knowledge regarding conjunctival inflammation grading methodologies and provides insights into novel developments in the field.

## 1. Introduction

Conjunctival hyperemia represents a complex inflammatory response characterized by microvascular dilation, mediated through the release of bioactive molecules including histamine, cytokines, and neuropeptides. Clinically observed as ocular redness, the dilation of conjunctival microvessels plays a critical role in the efferent component of the immune system, delivering both humoral and cellular immune components to the site of inflammation. The readily observable nature of the conjunctival microvasculature distinguishes it as a uniquely accessible circulatory system for clinical assessment, enabling non-invasive detection of both physiological variations and pathological processes [1]. This clinical finding accounts for the majority of non-refractive ocular complaints in healthcare settings [2].

Characterized by a reddish appearance of conjunctival tissue secondary to microvascular dilation, conjunctival hyperemia encompasses numerous etiologies that can be differentiated into infectious and non-infectious subtypes. The epithelial layer covering the conjunctiva acts as the initial barrier preventing infectious agents from entering. When this protective layer is compromised, susceptibility to infection increases. Complementary defense systems include antimicrobial tear film constituents (immunoglobulins and lysozyme), the conjunctival microvascular response, and mechanical clearance through the combined actions of blinking and tear flow [3].

Conjunctival hyperemia represents a frequent clinical finding across a spectrum of ocular disorders, encompassing conjunctivitis, uveitis, elevated intraocular pressure due to glaucoma, and ophthalmic side effects [4]. While ocular redness following antiglaucoma therapy is typically classified as a mild adverse effect, its significance increases substantially when analyzing medication compliance patterns. Patient complaints regarding topical medication-induced conjunctival hyperemia predominantly center on cosmetic concerns; however, this aesthetic dissatisfaction represents a significant barrier to optimal medication adherence and may compromise long-term therapeutic outcomes.

The accurate assessment and quantification of conjunctival inflammatory changes are essential for clinical decision-making and therapeutic monitoring. Historically, the evaluation of conjunctival inflammation has relied primarily on clinical observation and subjective grading by experienced practitioners. This approach, while practical and widely accessible, has been hampered by significant inter-observer and intra-observer variability, making it challenging to standardize assessments across different clinical settings and research protocols. The recognition of these limitations has driven the development of various grading scales aimed at providing more consistent and reproducible methods for quantifying conjunctival inflammation.

The clinical significance of accurate conjunctival inflammation assessment extends beyond simple diagnostic classification. In conditions such as dry eye disease, allergic conjunctivitis, contact lens-associated complications, and cicatrizing conjunctivitis, the ability to detect subtle changes in inflammatory status directly impacts treatment decisions and prognostic assessments [5]. Furthermore, the increasing emphasis on evidence-based medicine and the conduct of clinical trials necessitate precise and reproducible outcome measures for evaluating therapeutic interventions.

Among the four cardinal signs heralding inflammation (i.e., rubor, tumor, dolor, and calor), rubor (i.e., redness) of the bulbar conjunctiva is the most relevant driver of clinical judgment in cases of ocular surface disorders. Clinical evaluation of conjunctival injection may be judged using two strategies. One is chromaticity and the other is vessel morphology, such as the area occupied by vessels, number of vessels, or diameter and tortuosity of vessels in the region of interest (ROI). Based on the analysis presented by Curti et al. in 2021, it becomes evident that contemporary automated approaches to conjunctival hyperemia grading draw upon two complementary analytical dimensions [6]. Their image-processing pipeline integrates color-based descriptors (extracted from red-green-blue, RGB and hue-saturation-value, HSV color spaces) with vessel-related morphological features derived from segmented vascular networks. The authors further highlight that the vascular network provides some of the most informative structural cues for estimating hyperemia severity, while colorimetric features remain sensitive to illumination variability and therefore require appropriate normalization. Taken together, these methodological choices implicitly support the view that effective hyperemia assessment relies on both chromatic intensity information and morphological characterization of conjunctival vasculature, reinforcing the dual-paradigm framework increasingly recognized in the literature.

Historically, the evaluation of conjunctival inflammation has relied primarily on clinical observation and subjective grading by experienced practitioners. The recognition of these limitations has driven the development of various grading scales aimed at providing more consistent and reproducible methods for quantifying conjunctival inflammation.

Early attempts at standardization focused on creating photographic reference scales that could provide visual anchors for clinical grading. The advent of digital imaging technology has subsequently enabled the development of objective measurement systems that can quantify inflammatory parameters, thereby reducing subjective bias and improving reproducibility.

Contemporary approaches to conjunctival inflammation grading range from simple categorical scales suitable for routine clinical use to complex multi-parameter systems designed for research applications. The choice of grading system depends on the setting and the specific objectives. Understanding the strengths and limitations of different grading approaches is crucial for selecting appropriate assessment methods.

## 2. Traditional Grading Systems

Several traditional grading scales have been developed over the past decades to standardize the clinical assessment of conjunctival hyperemia. These include descriptive scales, exemplified by the slit-lamp findings classification scale and the Mandell scale for conjunctival injection, as well as photographic reference–based systems such as the McMonnies and Chapman-Davies (MC-D) scale, the Cornea and Contact Lens Research Unit scale (CCLRU scale) or Institute for Eye Research (IER) scale, the Efron scale, and the Validated Bulbar Redness (VBR) scale, all of which remain widely used benchmarks in clinical evaluation (Table 1). These scales have been extensively validated in both clinical practice and research settings, though each presents distinct advantages and limitations.

### 2.1. Descriptive Grading Scales

#### 2.1.1. Slit-Lamp Findings Classification Scale

In 1984, the Food and Drug Administration (FDA) developed the first standardized slit-lamp grading scale to systematically characterize conjunctival hyperemia observed during biomicroscopic examination, utilizing a numerical grading system complemented by specific descriptive parameters: 0 (None—No injection present), 1 (Trace—Slight limbal, bulbar and/or palpebral injection), 2 (Mild—Mild limbal, bulbar, and/or palpebral injection), 3 (Moderate—Significant, segmented limbal, diffuse bulbar or palpebral injection) and 4 (Severe—Severe limbal injection in the circumcorneal region, diffuse bulbar involving episcleral or scleral vessels, or palpebral injection) [1].

#### 2.1.2. Mandell Scale for Conjunctival Injection

In 1987, Mandell introduced a refined grading methodology for conjunctival injection derived from the FDA classification system, incorporating decimal subdivisions within the 0–4 integer scale to enable precise anatomical localization of conjunctival involvement: A. None—grade 0; B. Mild conjunctival hyperaemia (a) palpebral—grade 1.1, (b) palpebral and/or bulbar—grade 1.2, C. Mild circumcorneal injection—grade 1.3, D. Moderate conjunctival hyperaemia (a) palpebral—grade 2.1, (b) palpebral and/or bulbar—grade 2.2, E. Moderate circumcorneal injection—grade 2.3, F. Severe conjunctival hyperaemia (a) palpebral—grade 3.1, (b) palpebral and/or bulbar—grade 3.2, G. Severe circumcorneal injection—grade 3.3, H. Other (grade by severity as either 1.9, 2.9, 3.9, 4.9) [1].

### 2.2. Reference Image-Based Subjective Grading Scales

#### 2.2.1. McMonnies and Chapman-Davies Scale

C. W. McMonnies and A. Chapman-Davies developed the first systematic grading methodology in 1987 for conjunctival hyperemia evaluation across diverse contact lens wearing populations and controls, implementing hypertonic saline solution (20%) for controlled vasodilation induction and capturing sequential photographs of the inferior conjunctival region for comparative assessment against standardized reference images. This photographic reference approach, while practical and widely accessible, demonstrated acceptable inter-observer and intra-observer reliability within the original study, revealing statistically significant differences in hyperemic responses among hard lens, soft lens, and non-lens wearing groups; however, the scale’s broader clinical validity has been constrained by inherent subjectivity in reference image interpretation, exclusive anatomical focus on the inferior conjunctival quadrant, and considerable variability observed in subsequent applications across diverse clinical settings lacking standardized assessment protocols [1].

#### 2.2.2. Cornea and Contact Lens Research Unit (CCLRU) Scale

The Cornea and Contact Lens Research Unit (CCLRU) scale represents one of the earliest and most widely adopted standardized grading systems for conjunctival hyperemia. In 1993, the Cornea and Contact Lens Research Unit in Australia established a subjective classification system addressing contact lens-associated complications, encompassing bulbar redness, limbal redness, lid redness, lid roughness white light reflex, lid roughness fluorescein, corneal staining characteristics (including type, depth, and extent), and conjunctival staining. This scale was later renamed as the Institute for Eye Research (IER) or the Brian Holden Vision Institute grading scale [1]. Each of the nine different complications was graded into four different severity categories ranging from grade 1 (very slight) to grade 4 (severe) [7].

The CCLRU scale focuses primarily on bulbar conjunctival redness and has been extensively validated in contact lens research and clinical practice. Studies have demonstrated that the CCLRU scale provides reasonable inter-observer agreement when used by trained practitioners, though variability increases when grading is performed by less experienced observers [8].

#### 2.2.3. Efron Scale

In 1997, Professor Nathan Efron established a comprehensive grading system comprising 16 sets of artist-rendered illustrations as an alternative to photographic reference standards that address major anterior segment complications related to contact lens wear, including conjunctival hyperemia.

These complications include conjunctival redness, limbal redness, corneal neovascularization, epithelial microcysts, corneal oedema, corneal staining, conjunctival staining, papillary conjunctivitis, blepharitis, meibomian gland dysfunction, superior limbic keratoconjunctivitis, corneal infiltrates, corneal ulcer, endothelial polymegethism, endothelial blebs, corneal distortion, with severity stratification indicated through a traffic-light color scheme progressing from normal (Grade 0, green-framing) to severe (Grade 4, red-framing) presentations. Thus, conjunctival hyperemia is graded on a 0–4 scale, similar to the CCLRU scale [1,9,10].

The Efron scale has gained widespread acceptance in clinical practice and research due to its comprehensive nature and clearly illustrated standards. Validation studies have shown good correlation between Efron scale grades and objective measurements of conjunctival redness [11].

#### 2.2.4. Validated Bulbar Redness (VBR) Scale

In 2007 Schulz et al. introduced the validated bulbar redness (VBR) scale, a 100-point photographic grading methodology for objective conjunctival hyperemia quantification. Experimental hyperemia was induced through instillation of hypertonic saline (5%), with standardized photographic documentation of the recovering nasal conjunctival tissue obtained via a digital camera attached to a zoom photo slit lamp under controlled magnification, illumination, and gaze parameters. The integrated computer interface enabled systematic comparison of acquired images with the reference photographic scale [1,12].

Traditional grading systems have also been adapted for specific clinical contexts. In contact lens practice, specialized scales have been developed to assess lens-related complications, including conjunctival indentation, staining, and inflammatory responses [13].

Despite their widespread use, traditional subjective grading systems for conjunctival hyperemia demonstrate significant inter-observer variability. In a comparative study published in 2020, Huntjens et al. evaluated the consistency of clinical assessments using standard subjective scales versus an automated image-analysis system. While subjective grading achieved intraclass correlation coefficients (ICC) between 0.85 and 0.96, the automated method demonstrated substantially higher inter-observer agreement (ICC > 0.99), underscoring the inherent subjectivity of conventional scales and the potential benefit of integrating objective measurement approaches in clinical and research settings [14]. Factors contributing to this variability include differences in lighting conditions, image quality, observer training, and subjective interpretation of reference standards. Additionally, the categorical nature of traditional scales limits their sensitivity to detect small changes in inflammatory status, which may be clinically significant in certain contexts.

The practical application of traditional grading scales in clinical settings requires consideration of multiple factors that influence assessment accuracy. Studies have shown that optimal lighting conditions are crucial for reliable grading, with standardized illumination protocols recommended to minimize variability [6]. While subjective grading scales demonstrate significant variability between novice and experienced observers, with less experienced examiners showing greater deviation from expert assessments, objective automated grading methods effectively minimize these experience-dependent discrepancies and enhance clinical reliability [14]. This has led to the development of structured training programs aimed at improving inter-observer agreement through standardized interpretation of photographic references and systematic evaluation protocols.

## 3. Objective Measurement Systems and Digital Imaging

The limitations of subjective grading systems have led to the development of objective measurement techniques that can quantify conjunctival inflammation with greater precision and reproducibility. Digital imaging technology has been applied to capture and analyze conjunctival images through image processing techniques.

The evolution of objective assessment methods has progressed from simple color analysis to complex multi-parameter systems incorporating artificial intelligence. The integration of artificial intelligence and machine learning algorithms has further enhanced the sophistication of objective assessment, enabling the development of systems that can automatically classify inflammatory severity with accuracy comparable to or exceeding that of experienced clinicians.

Various technologies have been developed to address the inherent variability of subjective grading, each offering distinct measurement parameters, advantages, and limitations (Table 2).

Early objective systems focused on measuring the red-green ratio in digital images of the conjunctiva, based on the principle that increased vascularity results in higher red pixel content relative to the background conjunctival tissue [9]. These systems demonstrated improved reproducibility compared to subjective grading but were limited by variations in lighting conditions, camera settings, and image quality. Subsequent developments have incorporated color correction algorithms and standardized imaging protocols to address these limitations.

The CLAHE (Contrast Limited Adaptive Histogram Equalization) algorithm enhances local contrast in digital images, making subtle vascular patterns more visible and amenable to quantitative analysis. Studies have shown that CLAHE-processed images correlate well with subjective grading scales while providing superior reproducibility and the ability to detect smaller changes in inflammatory status [15].

Modern objective grading systems have evolved to incorporate multiple image analysis parameters beyond simple color analysis [18].

The integration of artificial intelligence and machine learning algorithms has further enhanced the sophistication of objective assessment, enabling the development of systems that can automatically classify inflammatory severity with accuracy comparable to or exceeding that of experienced clinicians.

The AOS (Automated Ocular Surface) grading system represents a commercially available objective assessment tool. This system uses proprietary algorithms to analyze digital images of the conjunctiva and provides numerical grades that correlate with traditional subjective scales. The 2022 study by Walker et al. demonstrates that the AOS^®^ Anterior objective grading system provides superior repeatability compared to traditional subjective grading methods. Both inter- and intra-subject repeatability of the objective system (0.15) was significantly better than the subjective methods (1.70). However, implementation of this objective grading approach requires specialized equipment and software. Image acquisition was performed utilizing the Keratograph K5 and Haag-Streit slit lamp. Following de-identification, images were imported into the AOS^®^ Anterior software for objective grading analysis. The AOS^®^ Anterior software (Sparca Corp, London, UK) is a commercial platform, necessitating institutional acquisition for implementation of this objective grading methodology. While this objective methodology demonstrates enhanced measurement precision and consistency, its clinical implementation necessitates investment in specialized imaging equipment and proprietary grading software [16]. In conclusion, while designated as an ‘automated’ system, the AOS^®^ Anterior grading method involves a semi-automated workflow requiring manual user input for region of interest (ROI) selection. Objective hyperemia assessment using the AOS^®^ Anterior system (Sparca Corp, London, UK) followed a standardized protocol: (1) digital image acquisition using; (2) image import into the AOS^®^ Anterior software platform; (3) manual delineation of the bulbar conjunctival region of interest by the examiner; (4) automated quantitative analysis of hyperemia parameters by proprietary algorithms. While the system requires manual ROI selection, subsequent analysis is fully automated and objective, eliminating subjective interpretation in the scoring process. This semi-automated approach introduces potential variability in ROI selection between operators, though the subsequent objective analysis demonstrates excellent repeatability and inter-observer agreement superior to fully subjective methods.

Three-dimensional imaging technologies have also been applied to conjunctival assessment. AS-OCT (anterior segment optical coherence tomography) is a noncontact imaging technology that produces detailed cross-sectional images using low-coherence interferometry in biological tissues, enabling precise visualization of the cornea, conjunctiva, sclera, anterior chamber, and adjacent anterior segment structures. Additionally, with the development of UHR-OCT, precise imaging of individual conjunctival layers is possible [17]. These advanced imaging modalities may be particularly valuable in research settings where comprehensive characterization of inflammatory changes is required.

The 2018 study by Akagi et al. established the feasibility of anterior segment optical coherence tomography angiography (AS-OCTA) for non-invasive visualization and quantitative assessment of conjunctival and intrascleral vasculature in healthy eyes. Employing swept-source OCT technology, the investigators demonstrated that AS-OCTA can successfully differentiate between superficial vascular layers (from the conjunctival epithelium to a depth of 200 μm) and deep vascular layers (from a depth of 200 μm to 1000 μm), with quantitative analysis including vessel density, vessel length density, vessel diameter index, and fractal dimension. The methodology exhibited high reproducibility and enabled detailed vascular mapping across different anatomical quadrants, with vessels in the nasal and temporal quadrants demonstrating greater density, reduced caliber, and increased pattern complexity compared to those in the inferior or superior quadrants. However, this pilot study was limited to 10 healthy subjects without ocular pathology. While the authors identified OCTA as a promising tool for evaluating conjunctival and intrascleral vasculatures relevant to ocular surface diseases the investigation did not address conjunctival hyperemia, inflammatory conditions, or pathological vascular changes. Thus, although this study provides important normative baseline data and technical validation, further investigation is warranted to establish the clinical applicability of AS-OCTA for assessing conjunctival inflammation and hyperemia in disease states [18].

Machine learning algorithms, particularly deep convolutional neural networks, have shown potential in providing objective, automated assessment of conjunctival hyperemia severity, addressing the limitations of subjective grading systems [4].

Despite their advantages, objective measurement systems face their own challenges and limitations. The requirement for specialized equipment and software may limit their accessibility in some clinical settings. Additionally, these systems may be sensitive to variations in image acquisition conditions, requiring standardized protocols and quality control measures to ensure reliable results. The interpretation of objective measurements may also require training and experience to correlate numerical values with clinical significance.

## 4. Specialized Grading Systems for Specific Conditions

The recognition that different inflammatory conditions may require specialized assessment approaches has led to the development of disease-specific grading systems. These specialized scales are designed to capture the unique features and clinical characteristics of particular conditions, providing more relevant and sensitive measures of disease activity and treatment response.

Cicatrizing conjunctivitis associated with ocular cicatricial pemphigoid represents a challenging condition requiring specialized assessment tools. Traditional grading scales may not adequately capture the progressive scarring and structural changes characteristic of this condition [20]. Novel grading systems for cicatrizing conjunctivitis incorporate multiple parameters beyond simple hyperemia assessment, providing a more comprehensive evaluation of disease severity and progression. The Foster classification system, one of the most frequently used staging systems, progresses from Stage I (conjunctivitis with subepithelial fibrosis) through Stage II (inferior fornix foreshortening) and Stage III (symblepharon formation) to Stage IV (end-stage disease with ankyloblepharon). More recently, Ong et al. developed a comprehensive clinical assessment tool that evaluates seven key components with moderate to excellent inter-observer agreement: inflammation grading (bulbar conjunctival hyperemia), scarring grading (upper and lower fornix symblephara, upper and lower central fornix depth measurement), and ocular morbidity grading (corneal vascularization, corneal opacity). Unlike traditional inflammation scoring schemes that relied predominantly on subjective assessments of conjunctival injection, this method employs comparison to a standard panel of photographs, demonstrating superior inter-observer agreement (intraclass correlation coefficient/ICC = 0.88, 95% confidence interval/CI 0.84–0.90). These systems provide standardized approaches for assessing conjunctival hyperemia within the context of cicatricial disease progression [21].

The development of specialized scales for allergic conjunctivitis has focused on incorporating specific features such as papillary hypertrophy, eosinophil infiltration, and seasonal variation patterns. These scales often include subjective symptom scores alongside objective clinical findings, recognizing the important relationship between patient-reported outcomes and clinical assessment [22]. Validation studies of allergic conjunctivitis scales have demonstrated their utility in clinical trials and therapeutic monitoring.

In dry eye disease the inflammatory changes may predominantly involve the conjunctival epithelium rather than the vasculature. Elevated expression of IFN-γ, IL-6, and MMP-9 in conjunctival epithelial cells, as reported by Yang et al. in 2019, correlates with conjunctival staining, highlighting epithelial inflammation as a central pathological feature [23]. A recent practical framework developed in Hong Kong highlights the importance of optimizing diagnostic and management strategies in dry eye disease, emphasizing tailored assessments that include not only symptom evaluation, but also objective measures of ocular surface inflammation. The study advocates for a standardized, risk-stratified approach to eye care that could facilitate the integration of automated grading systems—such as those for conjunctival hyperemia—into routine clinical workflows [24]. Yokoi et al. in 2019 proposed a tear-film-oriented diagnosis (TFOD) strategy for dry eye that emphasizes layer-by-layer analysis of the ocular surface and underscores the need for validated, stratified assessment tools for surface-disease evaluation [25].

Contact lens-related conjunctival complications have also been graded using specific scales to address the unique inflammatory patterns associated with lens wear. These scales consider factors such as mechanical trauma, hypoxic stress, and allergic sensitization, providing targeted assessment tools for contact lens practitioners [26]. The validation of these specialized scales has demonstrated their utility in identifying lens-related complications and guiding management decisions.

Infectious conjunctivitis presents another area where specialized grading may be beneficial. While traditional scales can assess general inflammatory severity, specialized systems for infectious conditions may incorporate parameters such as discharge character, lymphadenopathy, and membrane formation that are particularly relevant for diagnosing and monitoring bacterial, viral, or other infectious etiologies [27].

The development of pediatric-specific grading systems has recognized that conjunctival inflammation in children may present differently than in adults. These systems often incorporate age-appropriate assessment techniques and consider the unique challenges of examining pediatric patients, including cooperation limitations and developmental variations in conjunctival anatomy [28]. The challenge of assessing conjunctival inflammation in specific populations extends beyond pediatric patients to include elderly individuals and those with systemic diseases affecting ocular tissues. Shi et al. in 2019 have shown that bulbar conjunctival microcirculation (blood flow velocity) declines with age in healthy subjects, indicating age-related vascular changes in the conjunctiva [29]. Additionally, patients with systemic conditions may exhibit chronic baseline conjunctival changes that differ from acute inflammatory responses, requiring specialized assessment frameworks that distinguish between disease-related structural alterations and superimposed acute inflammation.

The development of condition-specific normal ranges and severity thresholds has emerged as an important area of research, with ongoing efforts to establish reference databases stratified by age, ethnicity, and underlying systemic conditions.

## 5. Validation Studies and Clinical Applications

The clinical utility of conjunctival inflammation grading scales depends on their validation through rigorous scientific studies. A comparative overview of performance characteristics across different grading approaches is presented in Table 3.

Although traditional subjective scales such as Efron and CCLRU have been widely used for assessing bulbar and palpebral conjunctival hyperemia, their repeatability and inter-observer agreement are limited. The 2020 study by Huntjens et al. demonstrated that the Automated Ocular Surface (AOS) grading method substantially improved repeatability (R^2^ = 0.998; Coefficient of Repeatability [CoR] 0.10–0.13) compared to Efron (R^2^ = 0.926; CoR 0.62) and CCLRU (R^2^ = 0.885–0.911; CoR 0.50–0.78). Similarly, inter-observer agreement, expressed as intraclass correlation coefficients (ICC), was markedly higher for the objective AOS method (>0.995) than for subjective scales (0.853–0.959), highlighting the enhanced reliability and sensitivity of automated grading systems in clinical and research applications [14].

The inter-observer agreement for subjective grading using the Efron scale was moderate to good, with ICCs of 0.80 [0.76–0.84] on the primary dataset and 0.75 [0.67–0.81] on an external validation set, reflecting the inherent variability between human observers in assessing conjunctival hyperemia. The semi-supervised AI model demonstrated strong concordance with human grading, achieving an intraclass correlation of 0.86 [0.76–0.93] between automatically segmented vessel density and ground truth annotations. Moreover, automated vessel density measurements correlated well with mean Efron scores (ρ = 0.83 for the test set and ρ = 0.80 for the external set), indicating that AI-based quantification can approximate clinician-assessed hyperemia with high reliability and reproducibility. These results highlight the potential of AI-based systems to reduce inter-observer variability and provide more objective assessment of conjunctival hyperemia in both clinical and research settings [30].

Reliability studies have consistently demonstrated that traditional subjective grading scales exhibit moderate to good intra-observer repeatability but more variable inter-observer agreement. These values indicate reasonable reliability for clinical use but highlight the potential for improvement through objective measurement approaches.

Comparative studies between subjective and objective grading methods have generally favored objective systems in terms of reproducibility. The 2025 study by Wong et al. demonstrated moderate inter-observer reproducibility for Efron grading (ICC 0.75–0.80), whereas a semi-supervised AI model showed higher reproducibility (ICC 0.86) and consistent repeatability, indicating that AI-assisted grading provides a more objective and reliable assessment of conjunctival hyperemia [30]. This improved reproducibility translates to enhanced ability to detect treatment effects in clinical trials and more precise monitoring of disease progression.

Studies investigating the responsiveness of grading scales for conjunctival hyperemia have shown that automated methods can better detect subtle changes than traditional ordinal scales. For example, Amparo et al. in 2017 demonstrated that the Ocular Redness Index (ORI) increased and decreased significantly after a surgical intervention, highlighting its high sensitivity to clinical change [31].

Sánchez Brea et al. in 2016 demonstrated that a computer-assisted grading system for bulbar conjunctival hyperemia, using an optimally defined region of interest (ROI), achieved intra-observer repeatability superior to that of human evaluators (mean difference < 0.19 units on the Efron scale) and produced measurements within the same range as expert assessments, indicating enhanced consistency and potential responsiveness to subtle clinical changes [32].

Peterson and Wolffsohn’s 2009 study demonstrated that digital image analysis of the anterior eye—combining vessel area and hue features—could account for between 69% and 98% of the variance in clinician-assigned grades on the Efron scale, thereby suggesting that objective grading methods can provide more sensitive and reliable assessments of ocular surface changes than traditional subjective grading alone [33].

The 2015 study by Ferrari et al. demonstrated that a semiautomated method for quantifying conjunctival redness—using Relative Redness Index (RRI) and Edge Feature (EF)—showed significant positive correlations with clinical Efron grading, patient-reported symptom severity (Visual Analog Scale, VAS), and conjunctival inflammatory cell counts. These findings indicate that this objective method is reproducible and reliably reflects both clinical severity and patient-perceived symptoms, highlighting the close relationship between measurable redness and subjective ocular discomfort [11].

The use of standardized grading scales has become increasingly recognized as a critical component for ensuring consistency and reliability in the clinical assessment of ocular surface signs. In 2015 Wolffsohn emphasizes that the consistent application of standardized grading scales for anterior eye signs, including bulbar hyperemia, is crucial for achieving reliable and reproducible documentation across clinical encounters. Such standardization not only enhances comparability of assessments between different practitioners but also strengthens the overall quality of patient monitoring, facilitates longitudinal evaluation, and supports more coherent and evidence-based decision-making in clinical settings where patients are managed by multiple clinicians [8].

The advent of digital ocular surface documentation has facilitated the incorporation of embedded grading tools, enabling precise longitudinal assessment and supporting evidence-based treatment decisions. In this context, Maqsood et al. in 2024 utilized AOS software to objectively quantify bulbar conjunctival redness and corneal epithelial defect size in patients treated with sutureless amniotic membrane, providing reproducible measurements of ocular surface inflammation over time [34]. Objective grading of hyperemia using AOS has consistently demonstrated greater repeatability compared to conventional subjective scales. Moreover, in pediatric blepharokeratoconjunctivitis, automated evaluation of conjunctival redness via the Redness Index correlated strongly with clinical grading, underscoring the applicability of digital assessment tools for standardized, long-term monitoring across patient populations as shown in the 2019 study conducted by Al-Hayouti et al. [19].

## 6. Technological Advances and Future Directions

The rapid advancement of digital technology and artificial intelligence has opened new possibilities for conjunctival inflammation assessment. Machine learning algorithms trained on large datasets of graded conjunctival images can achieve classification accuracy that matches or exceeds human experts while providing consistent and objective assessments [35]. These AI-based systems represent a significant step toward fully automated conjunctival grading that could be integrated into routine clinical workflows.

We recently developed a semi-supervised deep learning model to automatically grade conjunctival hyperemia from slit-lamp images. Our study, published in 2025 in the Annals of the New York Academy of Sciences, utilized limited labeled data supplemented with additional unlabeled images to train the model for conjunctival vessel segmentation and density calculation. Our results demonstrated strong correlation between model-derived vessel densities and manual Efron gradings, with performance comparable to inter-rater agreement among human experts. This approach demonstrates the potential for objective, automated assessment of conjunctival hyperemia in clinical practice, addressing the inherent subjectivity and variability of traditional manual grading methods [30].

Smartphone-based imaging systems have emerged as a promising approach for expanding access to objective conjunctival assessment. These systems leverage the high-quality cameras and computational power of modern smartphones to capture and analyze conjunctival images using dedicated applications. Validation studies of smartphone-based grading systems have demonstrated reasonable correlation with traditional assessment methods, suggesting potential for widespread clinical adoption [36]. Recently, Rodriguez et al. in 2025 developed and validated a smartphone-based automated system for grading conjunctival hyperemia in patients with dry eye disease [36]. Using EyeCup-acquired images and a conjunctival segmentation algorithm, the system quantifies redness intensity and vascular orientation, producing scores comparable to expert evaluation. In a multicenter study including 450 subjects and nearly 30,000 images, the system achieved a mean absolute error of 0.45 on a 0–4 scale, with 93% of predictions within one unit of expert scores. These findings indicate that smartphone-based automated grading provides a rapid, reproducible, and sensitive assessment of conjunctival hyperemia, offering significant advantages over traditional subjective methods by reducing inter-observer variability and improving standardization.

Advanced imaging modalities continue to expand the possibilities for conjunctival assessment. Binotti et al. in 2024 demonstrated that conjunctival vessel density measured by optical coherence tomography angiography (OCTA) is significantly associated with ocular surface inflammation, suggesting that OCTA could serve as a non-invasive, quantitative biomarker for monitoring inflammatory changes in the conjunctiva [37]. Unlike conventional imaging that captures only surface vessel appearance, OCTA provides depth-resolved imaging of conjunctival vasculature, enabling quantification of vessel density at different tissue layers [18]. OCTA conjunctival vessel density may be a novel, automated and objective surrogate for ocular surface inflammation that could detect subclinical inflammation. This technology has proven particularly valuable in detecting early microvascular alterations that may precede clinically apparent hyperemia, offering potential for earlier intervention in inflammatory conditions. The non-invasive nature of OCTA makes it especially suitable for longitudinal monitoring of treatment response in chronic inflammatory conditions.

Confocal microscopy enables cellular-level visualization of conjunctival tissues, potentially allowing for more precise characterization of inflammatory changes and monitoring of treatment response [38].

The integration of multiple assessment modalities into comprehensive evaluation platforms represents another promising direction. These systems combine traditional grading approaches with objective measurements, patient-reported outcomes, and biomarker analysis to provide holistic assessments of conjunctival health. Such integrated approaches may offer superior diagnostic accuracy and clinical utility compared to single-parameter assessment methods [5].

Telemedicine applications have highlighted the need for grading systems that can be effectively used in remote consultation settings. The development of standardized imaging protocols and automated analysis systems is essential for supporting teleophthalmology initiatives and expanding access to specialized care. Remote grading systems must balance the need for clinical accuracy with practical considerations such as equipment requirements and user training. In their 2018 study, Macchi et al. introduced a photographic bulbar redness grading scale and highlighted that the increased use of digital conjunctival photography facilitates remote assessment by centralized reading centres, thereby supporting the development of hyperemia grading systems suitable for tele-ophthalmology [9]. Cao et al. in 2023 highlighted that anterior-segment imaging devices hold promise for remote diagnosis and management of cornea and external ocular diseases [39]. Nevertheless, substantial gaps remain in the evidence regarding their diagnostic accuracy and inter-observer reliability, underscoring the need for rigorous validation studies to support their implementation in telemedicine.

The standardization of grading scales across different healthcare systems and countries remains an important goal for improving global eye care. International collaborative efforts are working to establish consensus standards for conjunctival inflammation assessment and to develop culturally appropriate adaptations of existing scales. These efforts are essential for facilitating international research collaboration and ensuring equitable access to high-quality eye care.

Personalized medicine approaches may require the development of individualized grading systems that account for patient-specific factors such as genetic background, environmental exposures, and comorbid conditions. Tse et al. in 2021 demonstrated that integrating clinical grading with proteomic data allows more precise assessment of ocular surface status than conventional clinical metrics [40].

The role of patient-reported outcome measures in conjunction with clinical grading scales continues to evolve. The 2017 study by Song H et al. demonstrated that patient-reported symptoms do not always correlate directly with clinical signs of inflammation, highlighting the need to combine objective clinical assessments (e.g., signs, imaging, grading) with subjective patient-reported outcomes. Their findings suggest that grading and evaluation methods for conditions such as conjunctival hyperemia should incorporate both objective indicators and patient experiences to enable a more comprehensive assessment [41]. The development of validated patient-reported outcome measures specific to conjunctival inflammation represents an important area for future research.

Environmental and occupational health applications of conjunctival grading scales are gaining recognition as tools for assessing exposure-related eye irritation and inflammation. These applications require specialized validation studies and may necessitate modifications to existing scales to account for exposure-specific inflammatory patterns and time courses. Findings from the prospective 2021 study of Chlasta-Twardzik et al. highlight the importance of considering environmental exposure when assessing conjunctival signs, suggesting that grading scales may need adaptation for occupational and environmental health applications [42].

The economic implications of different grading approaches are increasingly important considerations in healthcare decision-making. Cost-effectiveness analyses comparing subjective and objective grading methods must account for factors such as equipment costs, training requirements, time efficiency, and the clinical value of improved accuracy and reproducibility. These economic evaluations will influence the adoption and implementation of new grading technologies in different healthcare settings.

## 7. Conclusions

The assessment of conjunctival inflammation through standardized grading scales represents a critical component of modern practice and research. This review has highlighted the evolution from subjective, experience-based assessments to sophisticated objective measurement systems that leverage advanced imaging and computational technologies. The journey from simple categorical scales to complex multi-parameter systems reflects the increasing recognition of conjunctival inflammation as a multifaceted process requiring nuanced evaluation approaches.

Traditional subjective grading systems have provided the foundation for standardized conjunctival assessment and continue to serve important roles in clinical practice. These systems offer practical advantages including widespread availability, ease of use, and established clinical familiarity. However, their inherent limitations in terms of inter-observer variability and sensitivity to subtle changes have driven the development of more sophisticated assessment approaches. The evidence clearly demonstrates that while subjective scales remain clinically relevant and practical for routine use, they may not provide sufficient precision for research applications or sensitive monitoring of therapeutic interventions.

The emergence of objective measurement systems represents a paradigm shift toward more precise and reproducible conjunctival inflammation assessment. Digital imaging technologies, enhanced by sophisticated algorithms including CLAHE processing and artificial intelligence, have demonstrated superior reproducibility and sensitivity compared to traditional subjective approaches. These systems offer particular advantages in clinical research settings where precise quantification of inflammatory changes is essential for demonstrating treatment efficacy and regulatory approval. The commercial availability of systems such as AOS grading software indicates the maturation of objective assessment technologies and their readiness for broader clinical adoption.

Specialized grading systems for specific conditions have filled important gaps in the assessment of unique inflammatory patterns and disease characteristics. The development of scales tailored to cicatrizing conjunctivitis, allergic conjunctivitis, dry eye disease, and contact lens-related complications demonstrates the value of condition-specific assessment approaches. These specialized systems often provide more relevant and sensitive measures of disease activity compared to generic inflammation scales, highlighting the importance of matching assessment tools to specific clinical contexts.

The extensive validation research has provided crucial insights into the performance characteristics of different grading approaches and their appropriate applications. The consistent finding that objective systems offer superior reproducibility while subjective scales may better reflect clinically apparent changes suggests that optimal assessment strategies may involve complementary use of both approaches. The choice between subjective and objective grading should be guided by the specific clinical or research objectives, available resources, and required precision.

Looking toward the future, several trends and opportunities are shaping the evolution of conjunctival inflammation assessment. The integration of artificial intelligence and machine learning promises to deliver fully automated grading systems that combine the objectivity of digital analysis with the clinical insight of expert assessment. Smartphone-based imaging systems offer the potential to democratize access to objective grading capabilities, particularly in resource-limited settings. Advanced imaging modalities including OCT and confocal microscopy may provide new dimensions of inflammatory assessment that extend beyond traditional surface examination.

The standardization of grading scales across different healthcare systems and populations remains an important priority for improving global eye care quality and facilitating international research collaboration. Cross-cultural validation studies and adaptation of grading systems for different populations will be essential for ensuring equitable access to high-quality conjunctival assessment. The integration of patient-reported outcomes with clinical grading information represents another important direction for developing comprehensive evaluation approaches that capture both objective clinical findings and subjective patient experiences.

The economic considerations surrounding different grading approaches will increasingly influence adoption decisions in healthcare systems worldwide. Cost-effectiveness analyses that account for equipment costs, training requirements, and clinical value will guide the implementation of new grading technologies. The balance between accuracy, reproducibility, and practical feasibility will continue to shape the development and adoption of conjunctival inflammation assessment tools.

In conclusion, the field of conjunctival inflammation grading has evolved significantly and continues to advance through technological innovation and rigorous scientific validation. The current landscape offers a range of assessment options suitable for different clinical and research applications, from practical subjective scales for routine clinical use to sophisticated objective systems for precise research applications. The future promises even more advanced and accessible tools that will further improve our ability to assess, monitor, and treat conjunctival inflammatory conditions. The continued development and validation of grading scales will remain essential for advancing ophthalmic care and improving patient outcomes across diverse clinical settings and populations.

## Figures and Tables

**Table 1:** Traditional reference image-based subjective grading systems [1].

Scale Name	Development Period	Description	Characteristics	Limitations
McMonnies and Chapman-Davies (MC-D) scale	1987	6 reference photographs graded 0–5 for conjunctival redness	First grading scale	Limited sensitivity and restricted grading range for ocular redness
CCLRU scale	1993	4 reference photographs graded 1–4 for bulbar redness	Commonly used in clinical practice	Poor sensitivity at low redness levels; requires threshold for grade discrimination
Efron scale	1997	5 reference images graded 0–4 for conjunctival redness	Commonly used in clinical practice	Based on artist-rendered illustrations; poor sensitivity and high variability
Validated bulbar redness (VBR) scale	2007	5 reference photographs graded 10–90 (20-point increments) for ocular redness	Finer categorization and linear scale	Relies on human visual comparison to reference images

**Table 2:** Objective measurement systems.

System/Technology	Type	Measurement Parameters	Key Features	Advantages	Limitations
Red-Green Ratio Analysis [9]	Digital imaging	Color content analysis	Measures red pixel content relative to background conjunctival tissue	Improved reproducibility vs. subjective grading	Sensitive to lighting conditions, camera settings, image quality
CLAHE (Contrast Limited Adaptive Histogram Equalization) Algorithm [15]	Image processing algorithm	Local contrast enhancement; vascular pattern analysis	Makes subtle vascular patterns more visible for quantitative analysis	Superior reproducibility; detects smaller inflammatory changes; correlates well with subjective scales	Requires standardized protocols and image processing expertise
AOS (Automated Ocular Surface) System [16]	Commercial software	Manual selection of the region of interest (ROI) by the examiner, despite the ‘automated’ designation”	Proprietary algorithms for digital image analysis; provides numerical grades	Superior repeatability than the subjective methods	Requires specialized equipment and software; potential variability in ROI selection between operators
OCT (Optical Coherence Tomography) [17]	3D imaging	Conjunctival assessment	Layer-by-layer visualization with ultrahigh-resolution OCT (UHR-OCT)	Valuable for research applications	High cost; Limited accessibility; specialized equipment
OCTA (OCT Angiography) [18]	Advanced imaging	Detailed vascular visualization	Quantitative analysis	High reproducibility and enabled detailed vascular mapping across different anatomical quadrants	High cost; specialized training required; Limited availability
AI (Artificial Intelligence)/Machine Learning Systems [19]	Automated analysis	Automated classification	Trained on large datasets; Matches or exceeds human expert accuracy	Consistent, objective; Fully automated potential; Eliminates observer bias	Requires large training datasets; Validation needed

**Table 3:** Comparative performance characteristics of different grading approaches [14].

Assessment Method	Intra-Observer Repeatability (ICC)	Inter-Observer Agreement (ICC)	Coefficient of Repeatability
Traditional subjective scales (Efron, CCLRU)	Efron: 0.926 and CCLRU: 0.885–0.911	0.853–0.959	Efron: CoR (Coefficient of Repeatability) 0.62 and CCLRU: CoR 0.50–0.78
Objective digital analysis systems	Higher consistency (0.998)	Higher consistency (>0.995)	CoR 0.10–0.13

## Data Availability

Not applicable.

## Abbreviations

The following abbreviations are used in this manuscript:

CCLRU	Cornea and Contact Lens Research Unit
VBR	Validated bulbar redness
ROI	Region of interest
RGB	Red-green-blue
HSV	Hue-saturation-value
MC-D	McMonnies and Chapman-Davies
IER	Institute for Eye Research
FDA	Food and Drug Administration
ICC	Intraclass correlation coefficients
CLAHE	Contrast Limited Adaptive Histogram Equalization
AOS	Automated Ocular Surface
OCT	Optical coherence tomography
UHR-OCT	Ultrahigh resolution—Optical coherence tomography
OCTA	Optical coherence tomography angiography
AI	Artificial intelligence
AS-OCT	Anterior segment—Optical coherence tomography
AS-OCTA	Anterior segment—Optical coherence tomography angiography
CI	Confidence interval
IFN-γ	Interferon-gamma
IL-6	Interleukin-6
MMP-9	Matrixmetalloproteinase-9
TFOD	Tear-film-oriented diagnosis
CoR	Coefficient of Repeatability
ORI	Ocular Redness Index
RRI	Relative Redness Index
EF	Edge Feature
VAS	Visual Analog Scale
